# Supplementary material for: Formation of a β-barrel membrane protein is catalyzed by the interior surface of the assembly machine protein BamA
Source: eLife. 2019 Nov 14;8:e49787. doi: 10.7554/eLife.49787 (PMC6887485; doi:10.7554/eLife.49787)
Supplement: Supplementary file 1. [file elife-49787-supp1.docx]

**Supplementary File 1: List of strains used.**

| **Strains** | **Genotype and relevant features** | **Reference** |
| --- | --- | --- |
| MC4100 | F^−^ *araD139* Δ*(argF-lac)U169 rpsL150 relA1 flbB5301 deoC1 ptsF25 rbsR thi* | (Casadaban, 1976) |
| NR698 | MC4100 *lptD4213 (*Δ330-352) | (Wu et al., 2005) |
| NR1134 | NR754 Δ*lptD::kan* pACYC184::*lptD* (*lptD* depletion strain) | (Lee et al., 2018) |
| JCM166 | MC4100 *ara^r/-^* Δ(*λatt-lom*)::*bla* P_BAD_*yaeT araC* Δ*yaeT* | (Wu et al., 2005) |
| DEK1 | MC4100, *bamA*^E470G^ (*bamA*^E470G^ haploid strain) | This study |
| DH5α λ*pir* | *supE4* Δ*lacU169* (φ80 *lacZ*ΔM15) *hsdR17* *recA1* *endA1* *gyrA96* *thi-1* *relA1* λ*pir* | (Metcalf et al., 1994; Simon et al., 1983) |
