# Supplementary material for: Formation of a β-barrel membrane protein is catalyzed by the interior surface of the assembly machine protein BamA
Source: eLife. 2019 Nov 14;8:e49787. doi: 10.7554/eLife.49787 (PMC6887485; doi:10.7554/eLife.49787)
Supplement: Supplementary file 2. [file elife-49787-supp2.docx]

**Supplementary File 2: List of plasmids used**

| **Plasmids** | **Description** | **Reference** |
| --- | --- | --- |
| pZS21 | P_Ltet_-dependent expression vector | (Lutz and Bujard, 1997) |
| pZS21::*His-bamA* | Encodes full-length BamA with an N-terminal His_8_ tag | (Kim et al., 2007) |
| pZS21::*His-bamA^G655Am^* | pZS21::*His-bamA* with G655Amber | This study |
| pZS21::*His-bamA^S657Am^* | pZS21::*His-bamA* with S657Amber | This study |
| pZS21::*His-bamA^T659Am^* | pZS21::*His-bamA* with G659Amber | This study |
| pZS21::*His-bamA^N666Am^* | pZS21::*His-bamA* with N666Amber | This study |
| pZS21::*His-bamA^I668Am^* | pZS21::*His-bamA* with I668Amber | This study |
| pZS21::*His-bamA^N427Am^* | pZS21::*His-bamA* with N427Amber | This study |
| pZS21::*His-bamA^Y432Am^* | pZS21::*His-bamA* with Y432Amber | This study |
| pZS21::*His-bamA^T434Am^* | pZS21::*His-bamA* with T434Amber | This study |
| pZS21::*His-bamA^E435Am^* | pZS21::*His-bamA* with E435Amber | This study |
| pZS21::*His-bamA^S439Am^* | pZS21::*His-bamA* with S439Amber | This study |
| pZS21::*His-bamA^Q441Am^* | pZS21::*His-bamA* with Q441Amber | This study |
| pZS21::*His-bamA^Y468Am^* | pZS21::*His-bamA* with Y468Amber | This study |
| pZS21::*His-bamA^E470Am^* | pZS21::*His-bamA* with E470Amber | This study |
| pZS21::*His-bamA^L471Am^* | pZS21::*His-bamA* with L471Amber | This study |
| pZS21::*His-bamA^R488Am^* | pZS21::*His-bamA* with R488Amber | This study |
| pZS21::*His-bamA^F490Am^* | pZS21::*His-bamA* with F490Amber | This study |
| pZS21::*His-bamA^N492Am^* | pZS21::*His-bamA* with N492Amber | This study |
| pZS21::*His-bamA^F494Am^* | pZS21::*His-bamA* with F494Amber | This study |
| pZS21::*His-bamA^N501Am^* | pZS21::*His-bamA* with N501Amber | This study |
| pZS21::*His-bamA^T511Am^* | pZS21::*His-bamA* with T511Amber | This study |
| pZS21::*His-bamA^G528Am^* | pZS21::*His-bamA* with G528Amber | This study |
| pZS21::*His-bamA^G530Am^* | pZS21::*His-bamA* with G530Amber | This study |
| pZS21::*His-bamA^D569Am^* | pZS21::*His-bamA* with D569Amber | This study |
| pZS21::*His-bamA^K610Am^* | pZS21::*His-bamA* with K610Amber | This study |
| pZS21::*His-bamA^D614Am^* | pZS21::*His-bamA* with D614Amber | This study |
| pZS21::*His-bamA^T615Am^* | pZS21::*His-bamA* with T615Amber | This study |
| pZS21::*His-bamA^G655Am^* | pZS21::*His-bamA* with G655Amber | This study |
| pZS21::*His-bamA^S657Am^* | pZS21::*His-bamA* with S657Amber | This study |
| pZS21::*His-bamA^T659Am^* | pZS21::*His-bamA* with G659Amber | This study |
| pZS21::*His-bamA^N666Am^* | pZS21::*His-bamA* with N666Amber | This study |
| pZS21::*His-bamA^I668Am^* | pZS21::*His-bamA* with I668Amber | This study |
| pZS21::*His-bamA^Q803Am^* | pZS21::*His-bamA* with Q803Amber | This study |
| pZS21::*His-bamA^F804Am^* | pZS21::*His-bamA* with F804Amber | This study |
| pZS21::*His-bamA^N805Am^* | pZS21::*His-bamA* with N805Amber | This study |
| pZS21::*His-BamA^S439C^* | pZS21::*His-bamA* with S439C | This study |
| pZS21::*His-BamA^N666C^* | pZS21::*His-bamA* with N666C | This study |
| pTrc99a | P_trp/lac_-dependent expression vector | (Amann et al., 1988) |
| pTrc99a::*lptD-FLAG* | Encodes full-length LptD with a C-terminal FLAG_3_ tag | This study |
| pTrc99a::*lptD4213-FLAG* | Encodes LptD4213 with a C-terminal FLAG_3_ tag | This study |
| pTrc99a::*lptD4213-FLAG^L245C^* | pTrc99a::*lptD4213-FLAG* with L245C | This study |
| pTrc99a::*lptD4213-FLAG^H262C^* | pTrc99a::*lptD4213-FLAG* with H262C | This study |
| pTrc99a::*lptD4213-FLAG^Y291C^* | pTrc99a::*lptD4213-FLAG* with Y291C | This study |
| pTrc99a::*lptD4213-FLAG^A709C^* | pTrc99a::*lptD4213-FLAG* with A709C | This study |
| pTrc99a::*lptD4213-FLAG^E733C^* | pTrc99a::*lptD4213-FLAG* with E733C | This study |
| pTrc99a::*lptD4213-FLAG^N737C^* | pTrc99a::*lptD4213-FLAG* with N737C | This study |
| pTrc99a::*FLAG-lamB* | Encodes full-length LamB with a N-terminal FLAG_3_ tag | This study |
| pTrc99a::*FLAG-ompF* | Encodes full-length OmpF with a N-terminal FLAG_3_ tag | This study |
| pZS21::*lptD-His* | Encodes full-length LptD with a C-terminal His_8_ tag | (Lee et al., 2018) |
| pZS21::*lptD-His^A233Am^* | pZS21::*lptD-His* with A233Amber | This study |
| pZS21::*lptD-His^K234Am^* | pZS21::*lptD-His* with K234Amber | This study |
| pZS21::*lptD-His^Y235Am^* | pZS21::*lptD-His* with Y235Amber | This study |
| pZS21::*lptD-His^T237Am^* | pZS21::*lptD-His* with T237Amber | This study |
| pZS21::*lptD-His^T238Am^* | pZS21::*lptD-His* with T238Amber | This study |
| pZS21::*lptD-His^Y240Am^* | pZS21::*lptD-His* with Y240Amber | This study |
| pZS21::*lptD-His^Y244Am^* | pZS21::*lptD-His* with Y244Amber | This study |
| pZS21::*lptD-His^Y248Am^* | pZS21::*lptD-His* with Y248Amber | This study |
| pZS21::*lptD-His^I259Am^* | pZS21::*lptD-His* with I259Amber | This study |
| pZS21::*lptD-His^T260Am^* | pZS21::*lptD-His* with T260Amber | This study |
| pZS21::*lptD-His^P261Am^* | pZS21::*lptD-His* with P261Amber | This study |
| pZS21::*lptD-His^H262Am^* | pZS21::*lptD-His* with H262Amber | This study |
| pZS21::*lptD-His^Y263Am^* | pZS21::*lptD-His* with Y263Amber | This study |
| pZS21::*lptD-His^W272Am^* | pZS21::*lptD-His* with W272Amber | This study |
| pZS21::*lptD-His^E273Am^* | pZS21::*lptD-His* with E273Amber | This study |
| pZS21::*lptD-His^N274Am^* | pZS21::*lptD-His* with N274Amber | This study |
| pZS21::*lptD-His^E275Am^* | pZS21::*lptD-His* with E275Amber | This study |
| pZS21::*lptD-His^F276Am^* | pZS21::*lptD-His* with F276Amber | This study |
| pZS21::*lptD-His^L289Am^* | pZS21::*lptD-His* with L289Amber | This study |
| pZS21::*lptD-His^D290Am^* | pZS21::*lptD-His* with L290Amber | This study |
| pZS21::*lptD-His^Y291Am^* | pZS21::*lptD-His* with Y291Amber | This study |
| pZS21::*lptD-His^W311Am^* | pZS21::*lptD-His* with W311Amber | This study |
| pZS21::*lptD-His^L312Am^* | pZS21::*lptD-His* with L312Amber | This study |
| pZS21::*lptD-His^F313Am^* | pZS21::*lptD-His* with F313Amber | This study |
| pZS21::*lptD-His^Y314Am^* | pZS21::*lptD-His* with Y314Amber | This study |
| pZS21::*lptD-His^W315Am^* | pZS21::l*ptD-His* with W315Amber | This study |
| pZS21::*lptD-His^Y678Am^* | pZS21::*lptD-His* with Y678Amber | This study |
| pZS21::*lptD-His^Q684Am^* | pZS21::*lptD-His* with Q684Amber | This study |
| pZS21::*lptD-His^V685Am^* | pZS21::*lptD-His* with V685Amber | This study |
| pZS21::*lptD-His^Y704Am^* | pZS21::*lptD-His* with Y704Amber | This study |
| pZS21::*lptD-His^N708Am^* | pZS21::*lptD-His* with N708Amber | This study |
| pZS21::*lptD-His^M716Am^* | pZS21::*lptD-His* with M716Amber | This study |
| pZS21::*lptD-His^L717Am^* | pZS21::*lptD-His* with L717Amber | (Lee et al., 2018) |
| pZS21::*lptD-His^G718Am^* | pZS21::*lptD-His* with G718Amber | (Lee et al., 2018) |
| pZS21::*lptD-His^V719Am^* | pZS21::*lptD-His* with V719Amber | This study |
| pZS21::*lptD-His^Y721Am^* | pZS21::*lptD-His* with Y721Amber | (Lee et al., 2018) |
| pZS21::*lptD-His^Y726Am^* | pZS21::*lptD-His* with MY726Amber | (Lee et al., 2018) |
| pZS21::*lptD-His^I728Am^* | pZS21::*lptD-His* with I728Amber | This study |
| pZS21::*lptD-His^N737Am^* | pZS21::*lptD-His* with N737Amber | This study |
| pZS21::*lptD-His^W739Am^* | pZS21::*lptD-His* with W739Amber | This study |
| pZS21::*lptD-His^K743Am^* | pZS21::*lptD-His* with K743Amber | This study |
| pZS21::*lptD-His^V747Am^* | pZS21::*lptD-His* with V747Amber | This study |
| pZS21::*lptD-His^Y748Am^* | pZS21::*lptD-His* with Y748Amber | This study |
| pZS21::*lptD-His^D749Am^* | pZS21::*lptD-His* with D749Amber | This study |
| pZS21::*lptD-His^I752Am^* | pZS21::*lptD-His* with I752Amber | This study |
| pZS21::*lptD-His^F754Am^* | pZS21::*lptD-His* with F754Amber | This study |
| pZS21::*lptD-His^N755Am^* | pZS21::*lptD-His* with N755Amber | This study |
| pZS21::*lptD4213-His* | Encodes LptD4213 (Δ330-352) with a C-terminal His_8_ tag | (Lee et al., 2018) |
| pZS21::*lptD4213-His^A233Am^* | pZS21::*lptD4213-His* with A233Amber | This study |
| pZS21::*lptD4213-His^K234Am^* | pZS21::*lptD4213-His* with K234Amber | This study |
| pZS21::*lptD4213-His^Y235Am^* | pZS21::*lptD4213-His* with Y235Amber | This study |
| pZS21::*lptD4213-His^T237Am^* | pZS21::*lptD4213-His* with T237Amber | This study |
| pZS21::*lptD4213-His^T238Am^* | pZS21::*lptD4213-His* with T238Amber | This study |
| pZS21::*lptD4213-His^Y240Am^* | pZS21::*lptD4213-His* with Y240Amber | This study |
| pZS21::*lptD4213-His^Y244Am^* | pZS21::*lptD4213-His* with Y244Amber | This study |
| pZS21::*lptD4213-His^Y248Am^* | pZS21::*lptD4213-His* with Y248Amber | This study |
| pZS21::*lptD4213-His^I259Am^* | pZS21::*lptD4213-His* with I259Amber | This study |
| pZS21::*lptD4213-His^T260Am^* | pZS21::*lptD4213-His* with T260Amber | This study |
| pZS21::*lptD4213-His^P261Am^* | pZS21::*lptD4213-His* with P261Amber | This study |
| pZS21::*lptD4213-His^H262m^* | pZS21::*lptD4213-His* with H262Amber | This study |
| pZS21::*lptD4213-His^Y263Am^* | pZS21::*lptD4213-His* with Y263Amber | This study |
| pZS21::l*ptD4213-His^R266Am^* | pZS21::*lptD4213-His* with R266Amber | This study |
| pZS21::*lptD4213-His^N269Am^* | pZS21::*lptD4213-His* with N269Amber | This study |
| pZS21::*lptD4213-His^W272Am^* | pZS21::*lptD4213-His* with W272Amber | This study |
| pZS21::*lptD4213-His^E273Am^* | pZS21::*lptD4213-His* with E273Amber | This study |
| pZS21::*lptD4213-His^N274Am^* | pZS21::*lptD4213-His* with N274Amber | This study |
| pZS21::*lptD4213-His^E275Am^* | pZS21::*lptD4213-His* with E275Amber | This study |
| pZS21::*lptD4213-His^F276Am^* | pZS21::*lptD4213-His* with F276Amber | This study |
| pZS21::l*ptD4213-His^L279Am^* | pZS21::*lptD4213-His* with L279Amber | This study |
| pZS21::l*ptD4213-His^L286Am^* | pZS21::*lptD4213-His* with L286Amber | This study |
| pZS21::*lptD4213-His^L289Am^* | pZS21::*lptD4213-His* with L289Amber | This study |
| pZS21::*lptD4213-His^D290Am^* | pZS21::*lptD4213-His* with L290Amber | This study |
| pZS21::*lpt4213D-His^Y291Am^* | pZS21::*lptD4213-His* with Y291Amber | This study |
| pZS21::*lptD4213-His^W311Am^* | pZS21::*lptD4213-His* with W311Amber | This study |
| pZS21::*lptD4213-His^L312Am^* | pZS21::*lptD4213-His* with L312Amber | This study |
| pZS21::*lptD4213-His^F313Am^* | pZS21::*lptD4213-His* with F313Amber | This study |
| pZS21::*lptD4213-His^Y314Am^* | pZS21::*lptD4213-His* with Y314Amber | This study |
| pZS21::*lptD4213-His^W315Am^* | pZS21::*lptD4213-His* with W315Amber | This study |
| pZS21::*lptD4213-His^Y678Am^* | pZS21::*lptD4213-His* with Y678Amber | This study |
| pZS21::*lptD4213-His^Q684Am^* | pZS21::*lptD4213-His* with Q684Amber | This study |
| pZS21::*lptD4213-His^V685Am^* | pZS21::*lptD4213-His* with V685Amber | This study |
| pZS21::*lptD4213-His^Y704Am^* | pZS21::*lptD4213-His* with Y704Amber | This study |
| pZS21::*lptD4213-His^N708Am^* | pZS21::*lptD4213-His* with N708Amber | This study |
| pZS21::*lptD4213-His^M716Am^* | pZS21::*lptD4213-His* with M716Amber | This study |
| pZS21::*lptD4213-His^L717Am^* | pZS21::*lptD4213-His* with L717Amber | (Lee et al., 2018) |
| pZS21::*lptD4213-His^G718Am^* | pZS21::*lptD4213-His* with G718Amber | (Lee et al., 2018) |
| pZS21::*lptD4213-His^V719Am^* | pZS21::*lptD4213-His* with V719Amber | This study |
| pZS21::*lptD4213-His^Y721Am^* | pZS21::*lptD4213-His* with Y721Amber | (Lee et al., 2018) |
| pZS21::*lptD4213-His^Y726Am^* | pZS21::*lptD4213-His* with MY726Amber | (Lee et al., 2018) |
| pZS21::*lptD4213-His^I728Am^* | pZS21::*lptD4213-His* with I728Amber | This study |
| pZS21::*lptD4213-His^N737Am^* | pZS21::*lptD4213-His* with N737Amber | This study |
| pZS21::*lptD4213-His^W739Am^* | pZS21::*lptD4213-His* with W739Amber | This study |
| pZS21::*lptD4213-His^K743Am^* | pZS21::*lptD4213-His* with K743Amber | This study |
| pZS21::*lptD4213-His^V747Am^* | pZS21::*lptD4213-His* with V747Amber | This study |
| pZS21::*lptD4213-His^Y748Am^* | pZS21::*lptD4213-His* with Y748Amber | This study |
| pZS21::*lptD4213-His^D749Am^* | pZS21::*lptD4213-His* with D749Amber | This study |
| pZS21::*lptD4213-His^I752Am^* | pZS21::*lptD4213-His* with I752Amber | This study |
| pZS21::*lptD4213-His^F754Am^* | pZS21::*lptD4213-His* with F754Amber | This study |
| pZS21::*lptD4213-His^N755Am^* | pZS21::*lptD4213-His* with N755Amber | This study |
| pZS21::*lptD4213-His^F276Am, N274I^* | pZS21::*lptD4213-His* with F276Amber and N274I | This study |
| pZS21::*lptD^ΔD330^-His^Y244Am^* | pZS21::*lptD ^ΔD330^-His* with Y244Amber | This study |
| pET23/42 | pET23a(+) with multiple cloning sites of pET42a(+), P_T7_-dependent expression vector | (Wu et al., 2005) |
| pET23/42::*lptD* | Encodes full-length LptD | (Chng et al., 2010) |
| pET23/42::*lptD^N274I^* | pET23/42::*lptD* with N274I | This study |
| pET23/42::*lptD4213* | Encodes LptD4213 (Δ330-352) | This study |
| pET23/42::*lptD4213^N274I^* | pET23/42::*lptD4213* with N274I | This study |
| pET23/42::*lptD*^Δ^*^D330^* | pET23/42::*lptD* with ΔD330 | This study |
| pET23/42::*lptD*^Δ^*^D330, N274I^* | pET23/42::*lptD*_Δ_*_D330_* with N274I | This study |
| pET23/42::*lptD-FLAG* | Encodes full-length LptD with a C-terminal FLAG_3_ tag | (Chng et al., 2012) |
| pET23/42::*lptD4213-FLAG* | Encodes LptD4213 (Δ330-352) with a C-terminal FLAG_3_ tag | This study |
| pET23/42::*lptD*^Δ^*^D330^-FLAG* | pET23/42::*lptD-FLAG* with ΔD330 | This study |
| pCDFDuet | P_T7_-dependent dual expression vector | Novagen |
| pCDF::*bamA* | Encodes full-length BamA | (Lee et al., 2018) |
| pCDF::*bamA^S436P^* | pCDF::*bamA* with S436P | This study |
| pCDF::*bamA^Q441R^* | pCDF::*bamA* with Q441R | This study |
| pCDF::*bamA^E470G^* | pCDF::*bamA* with E470G | This study |
| pCDF::*bamA^D512G^* | pCDF::*bamA* with D512G | This study |
| pDS132 | *R6K* *ori*, *mobRP4*, *sacB*, Cam^R^ | (Philippe et al., 2004) |
| pDS132::*bamA* | Encodes full-length BamA | This study |
| pDS132::*bamA^E470G^* | pDS132::*bamA* with E470G | This study |

**Supplementary References:**

1. Amann E, Ochs B, Abel KJ. 1988. Tightly regulated tac promoter vectors useful for the expression of unfused and fused proteins in Escherichia coli. *Gene* **69**:301–315. doi:10.1016/0378-1119(88)90440-4
2. Chng S-S, Ruiz N, Chimalakonda G, Silhavy TJ, Kahne D. 2010. Characterization of the two-protein complex in Escherichia coli responsible for lipopolysaccharide assembly at the outer membrane. *Proceedings of the National Academy of Sciences of the United States of America* **107**:5363–8. doi:10.1073/pnas.0912872107
3. Chng S-S, Xue M, Garner RA, Kadokura H, Boyd D, Beckwith J, Kahne D. 2012. Disulfide rearrangement triggered by translocon assembly controls lipopolysaccharide export. *Science (New York, NY)* **337**:1665–8. doi:10.1126/science.1227215
4. Kim S, Malinverni JC, Sliz P, Silhavy TJ, Harrison SC, Kahne D. 2007. Structure and function of an essential component of the outer membrane protein assembly machine. *Science (New York, NY)* **317**:961–4. doi:10.1126/science.1143993
5. Lee J, Sutterlin HA, Wzorek JS, Mandler MD, Hagan CL, Grabowicz M, Tomasek D, May MD, Hart EM, Silhavy TJ, Kahne D. 2018. Substrate binding to BamD triggers a conformational change in BamA to control membrane insertion. *Proceedings of the National Academy of Sciences of the United States of America* **115**:2359–2364. doi:10.1073/pnas.1711727115
6. Lutz R, Bujard H. 1997. Independent and tight regulation of transcriptional units in Escherichia coli via the LacR/O, the TetR/O and AraC/I1-I2 regulatory elements. *Nucleic acids research* **25**:1203–10.
7. Philippe N, Alcaraz J-P, Coursange E, Geiselmann J, Schneider D. 2004. Improvement of pCVD442, a suicide plasmid for gene allele exchange in bacteria. *Plasmid* **51**:246–255. doi:10.1016/j.plasmid.2004.02.003
8. Wu T, Malinverni J, Ruiz N, Kim S, Silhavy TJ, Kahne D. 2005. Identification of a multicomponent complex required for outer membrane biogenesis in Escherichia coli. *Cell* **121**:235–245.
